# Telomere Maintenance and DNA Repair: A Bidirectional Relationship in Cancer Biology and Therapy

**DOI:** 10.3390/cancers17142284

**Published:** 2025-07-09

**Authors:** Nina Rembiałkowska, Mikołaj Sędzik, Monika Kisielewska, Wiktoria Łuniewska, Kamil Sebastianka, Klaudia Molik, Katarzyna Skinderowicz, Jacek Kuźnicki, Joanna Tunikowska, Julita Kulbacka

**Affiliations:** 1 Department of Molecular and Cellular Biology, Faculty of Pharmacy, Wroclaw Medical University, Borowska 211A, 50-556 Wroclaw, Poland; julita.kulbacka@umw.edu.pl; 2 Faculty of Medicine, Wroclaw Medical University, Pasteura 1, 50-367 Wroclaw, Poland; mikolaj.sedzik@student.umw.edu.pl (M.S.); monika.kisielewska@student.umw.edu.pl (M.K.); wiktoria.luniewska@student.umw.edu.pl (W.Ł.); kamil.sebastianka@student.umw.edu.pl (K.S.); klaudia.molik@student.umw.edu.pl (K.M.); katarzyna.skinderowicz@student.umw.edu.pl (K.S.); jacek.kuznicki@student.umw.edu.pl (J.K.); 3 Faculty of Veterinary Medicine, Wroclaw University of Environmental and Life Sciences, 50-375 Wroclaw, Poland; joanna.tunikowska@upwr.edu.pl; 4 Department of Immunology and Bioelectrochemistry, State Research Institute Centre for Innovative Medicine, Santariškių 5, 08410 Vilnius, Lithuania

**Keywords:** telomere maintenance, genomic instability, telomerase activation, alternative lengthening of telomeres, DNA repair pathways, telomere-targeted cancer therapy

## Abstract

Telomeres are repetitive DNA sequences at the ends of chromosomes that protect against genomic instability and prevent unwanted DNA damage responses. In most somatic cells, telomeres progressively shorten with each division, limiting cellular lifespan. However, cancer cells bypass this limitation by activating telomerase or the alternative lengthening of telomeres, enabling unchecked proliferation and tumor progression. This review examines the molecular mechanisms underlying telomere maintenance and their intricate relationship with DNA repair pathways. We discuss how telomere-associated proteins regulate genomic stability and explore therapeutic strategies targeting telomerase and alternative lengthening of telomeres. Challenges such as resistance mechanisms and off-target effects are also considered, highlighting the need for precision approaches in telomere-based cancer therapies.

## Simple Summary

Cancer cells manage to live longer and grow uncontrollably by protecting the ends of their chromosomes, known as telomeres. In healthy cells, telomeres naturally shorten over time, leading to aging and death of the cell. But in cancer, special mechanisms keep these telomeres from shrinking, allowing the cells to avoid death and continue dividing. This review explains how cancer cells achieve this and the relationship between telomere protection and the systems that repair damaged DNA. The study also explores new treatment approaches that aim to block these processes in cancer cells. These include drugs, immune therapies, and even natural substances that target the proteins or structures involved in telomere maintenance. While some treatments are still being tested in early trials, others have already shown promise in specific cancers. The authors also highlight the challenges of targeting telomeres without harming normal cells, and how telomere length and behavior can help doctors predict treatment success or detect cancer earlier. This work is important because it may lead to better, more personalized treatments and help fight cancer more effectively in the future.

## 1. Introduction

**Figure 1:**
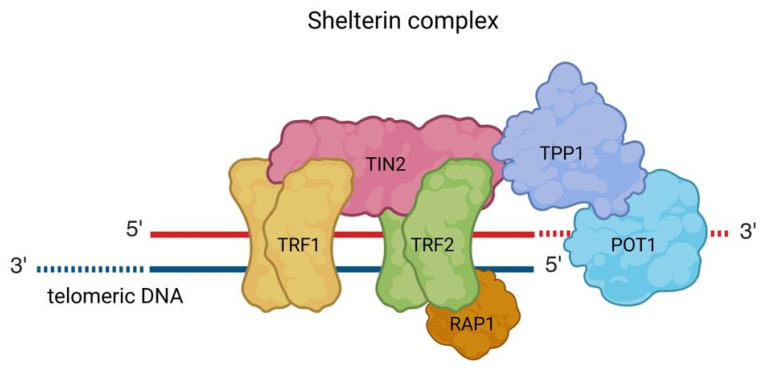
Human shelterin complex consists of six proteins: TRF1, TRF2, RAP1, TIN2, TPP1, and POT1. The shelterin complex interacts with both dsDNA and ssDNA regions of telomeric DNA. TRF1 and TRF2 form homodimers that attach specifically to the double-stranded segments, while POT1 binds to the single-stranded portions of the telomere.

Telomeres are repetitive DNA sequences that form the ends of eukaryotic chromosomes, with the specific repeat unit varying between species. For instance, the hexameric sequence (5′-TTAGGG-3′) is characteristic of vertebrates, including humans [1]. They are localized at the end of each chromosome arm in the form of a T-loop and a D-loop. A six-subunit protein complex-shelterin-stabilises the whole spatial structure and allows cells to distinguish telomeres from sites of DNA damage. Shelterin protein complex consists of six proteins: telomeric repeat-binding factor 1 (TRF1), telomeric repeat binding factor 2 (TRF2), repressor/activator protein 1 (RAP1), TRF1-interacting nuclear factor 2 (TIN2), tripeptidyl-peptidase (TPP1) and protection of telomeres protein 1 (POT1), as shown in Figure 1. The shelterin complex interacts with both the double-stranded (dsDNA) and single-stranded (ssDNA) regions of telomeric DNA. TRF1 and TRF2 form homodimers that attach specifically to the double-stranded segments, while POT1 binds to the single-stranded portions of the telomere. This prevents inappropriate DNA repair at telomeric regions and damage response pathway activation [2,3,4,5].

**Figure 2:**
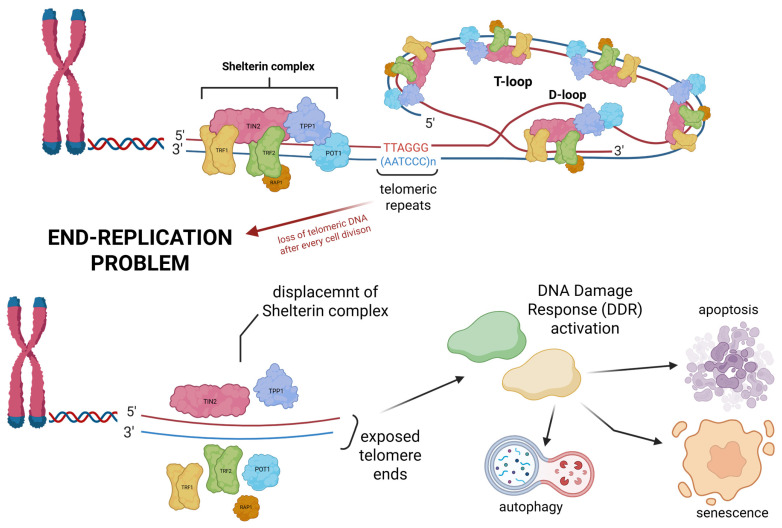
The schematic illustrates telomeres, repetitive DNA sequences, located at the ends of chromosomes. Shelterin complex, composed of TRF1, TRF2, TIN2, TPP1, POT1, and RAP1, stabilizes the whole spatial structure and protects chromosome ends. Telomeric DNA forms a characteristic T-loop structure, within which a D-loop is embedded, enabling the telomere to fold back on itself and further shield the DNA ends from being recognized as damaged. After each cell division, a portion of telomeric DNA is gradually lost, leading to the “end-replication problem”. When telomeres become critically short, the shelterin complex is displaced, leading to exposed telomere ends. These are recognized as DNA damage and trigger a DDR, which may result in apoptosis, senescence, or autophagy.

Telomeres act as a “buffer” that ensures the completion of the replication process, thus helping to maintain genomic stability. Telomeres delay DNA loss during replication at the 5′ end of the lagging strand when the RNA primer is removed due to the DNA polymerase’s ability to synthesise new strands only in the 5′ to 3′ direction. Noteworthily, it means that after every cell division, somatic cells inevitably lose a part of telomeric DNA (about 200 nucleotides), thereby losing their ability to proliferate at some point (“end-replication problem” phenomenon). The gradual loss of telomeric tandem repeats leads to the displacement of shelterin proteins, destabilizing the t-loop structure and exposing telomere ends. This exposure is recognized by the DNA damage response (DDR) machinery, which interprets it as a double-strand break (DSB). Consequently, DDR is activated, triggering downstream pathways that drive cells toward senescence, apoptosis, or autophagy as shown in Figure 2 [2,3,6,7,8,9,10,11]. While this process is associated with aging and disease, it is a normal part of the cell cycle, and its disruption may result in pathological outcomes [12].

At birth, the length of telomeres varies between inherited paternal and maternal chromosomes as well as between individuals. These differences stay stable through the cell’s lifetime [13]. Apart from the progressive erosion of telomeres in normal human cells, some telomeres tend to shorten faster than others, which is caused by large deletions at certain telomeres, local processing dynamics, tissue-level variation, and damage-related influences. Telomerase preferentially elongates shorter telomeres, promoting a more homogeneous distribution of telomere length [14]. Studies using sensitive single telomere length analysis have shown that some telomeres in normal, telomerase-negative human fibroblasts become nearly devoid of telomeric repeats at senescence [13].

Cancer cell survival, proliferation, and metastatic potential are driven by a multitude of distinct molecular mechanisms [15]. Currently, cancer continues to be a major global health concern, frequently cited as the second leading cause of death worldwide [16,17,18]. A critical mechanism contributing to the extended lifespan of cancer cells is the active maintenance of telomere length achieved by telomerase upregulation or alternative lengthening of telomeres (ALT). Telomeres in cancer cells tend to be significantly shorter than in normal cells, though their lengths can sometimes be extended or inconsistent [19]. In some cancer cells containing telomerase, critically short ‘T-stump’ telomeres, which have lost nearly all of their telomeric sequence, are found. T-stumps preserve their function as a basic telomeric protective structure as they still bind telomere-associated proteins like TRF1 and TRF2. High telomerase activity and lack of checkpoint pathways allow cells to tolerate these minimal telomeric structures (t-stumps) and continue dividing despite telomere erosion [20]. The proportion of such telomeres correlates with unfavorable clinical outcomes of first-line ABVD therapy, which is a chemotherapy regimen including Adriamycin (doxorubicin), Bleomycin, Vinblastin, and Dacarbazin, used in patients with Hodgkin Lymphoma (HL) [21].

In normal cells, the arrangement of telomeres within the cell changes depending on what stage of the cell cycle the cell is in. Telomeres are widely dispersed throughout the nucleus in the G0/G1 and S phases [22]. During the late G2 phase, telomeres assume a telomeric disk consisting of non-overlapping telomeres. In cancer cells, instead of forming the organized telomeric disk, telomeres create aggregates. Such aggregates are clusters of telomeres located close together that cannot be distinguished as separate due to the 200 nm limit of optical resolution [23]. This can also be influenced by the deregulated expression of the c-Myc protein, which induces telomere aggregates, fusions, and breakage-fusion-bridge cycles. During these cycles, chromosome ends fuse together and form dicentric chromosomes. During anaphase, these dicentric chromosomes are pulled apart and break, creating telomere-free chromosome ends that can fuse again with other chromosomes and repeat the cycle through successive cell divisions [24,25]. This causes permanent changes in the structure of chromosomes, leading to genomic instability [23].

Numerous telomere-associated proteins have been demonstrated to regulate DNA repair processes and prevent chromosomal end fusions. Shelterin safeguards telomere integrity by distinguishing natural chromosome ends from DNA breaks. It prevents harmful DNA repair and damage response pathway activation, while also recruiting telomerase to address the end-replication problem [4,26,27]. In Epstein–Barr Virus (EBV)-associated HL, we can observe how disruption of the shelterin complex can lead to cancer development. The oncogenic EBV protein LMP1 expression induces a substantial downregulation at both the transcriptional and translational levels of key telomeric proteins that are components of the shelterin complex [28].

Given the potential for telomere-targeted therapies in diverse cancers, this study reviews critical molecular mechanisms that facilitate telomere length maintenance [29]. In this article, we begin by describing telomerase upregulation and ALT, which is a telomerase-independent telomere maintenance mechanism [30]. Since ALT-positive tumors are aggressive and treatment-resistant, developing effective and selective therapies is urgently needed [29]. This article reviews the potential therapeutic targets and strategies designed to affect these mechanisms. We have focused on the influence of DNA repair complexes and addressed the challenges inherent in targeting telomere dynamics for cancer therapy. We explore the implications of off-target effects, compensatory mechanisms, and the utilization of biomarkers to guide therapeutic interventions.

## 2. Mechanisms of Telomere Maintenance in Cancer

Cancer cells can maintain telomeres by either telomerase upregulation or ALT. Around 85–90% of cancers use telomerase to attain unlimited replicative potential, whereas the remaining 10–15% involve homologous recombination (HR) mechanisms for preserving telomere length [2,3,6,9].

**Figure 3:**
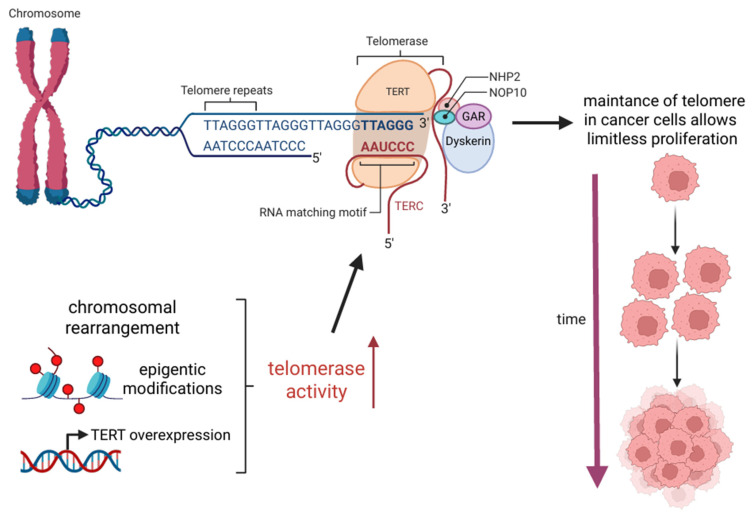
The schematic illustrates the mechanism of telomere maintenance in cancer cells resulting from telomerase upregulation. Telomerase is a ribonucleoprotein complex composed of telomerase reverse transcriptase, the telomerase RNA component, and associated proteins including dyskerin, NHP2, NOP10, and GAR1. TERC provides the RNA template required by TERT to synthesize telomeric DNA repeats, thereby elongating shortened telomeres. In cancer cells, telomerase activity is frequently upregulated through TERT overexpression, epigenetic modifications, as well as chromosomal rearrangements. This enhanced telomerase activity enables continuous telomere elongation, allowing cells to bypass replicative senescence and acquire limitless proliferative potential.

Telomerase is a complex enzyme composed of multiple subunits, each playing a crucial role in its function. Its core components include the catalytic subunit telomerase reverse transcriptase (TERT) and human telomerase RNA (TERC), which serves as a template for synthesizing new telomeric DNA. Additionally, several associated proteins are required for the proper assembly and stabilization of its spatial structure, including dyskerin, reptin, pontin, NOP10, NHP2, and GAR1. Telomerase functions by reverse transcribing the telomeric RNA template, primarily synthesizing the “canonical” TTAGGG hexanucleotide sequence, although occasional variations may occur [2,3]. Neoplastic cells most commonly achieve TERT overexpression due to genetic alterations in the TERT promoter or TERT gene [2,11]. Moreover, epigenetic modifications, including TERT promoter methylation, might also contribute to telomerase upregulation, which was found in many cancer types, among others, in melanomas, thyroid cancers, brain cancers, urinary cancers, and hepatocellular carcinomas [2,10,31,32,33,34]. Furthermore, in B-cell neoplasms, TERT upregulation has been observed as a consequence of chromosomal translocation, where the TERT gene is relocated near immunoglobulin gene loci. This repositioning places TERT under the control of strong immunoglobulin gene promoters, leading to increased TERT transcription and enhanced telomerase activity. Interestingly, TERT overexpression is not always a direct consequence of genomic alterations. Instead, it can be regulated by various transcriptional activators and signalling pathways. Notably, factors such as MYC, NF-κB, and β-catenin have been identified as key regulators that enhance TERT transcription, thereby increasing telomerase levels within the cell [2]. Telomerase reactivation represents a fundamental hallmark of cancer. By sustaining telomere integrity, cancer cells can bypass replicative senescence and apoptosis, acquiring the capacity for limitless proliferation, an essential feature of malignant transformation and tumor progression [35]. The telomerase-dependent mechanism of telomere maintenance is illustrated in Figure 3.

**Figure 4:**
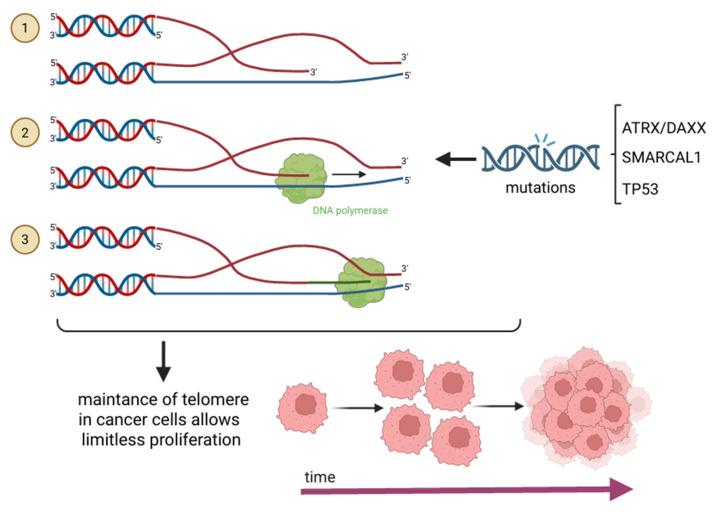
The ALT pathway enables telomere maintenance in the absence of telomerase through an HR-based mechanism. ① The 3′ single-stranded G-overhang of one telomere invades the double-stranded telomeric region of another chromosome, forming a displacement loop (D-loop) structure that resembles a replication fork. ② This intermediate is recognized by DNA polymerase, which extends the invading strand using the homologous telomeric DNA as a template. ③ The process results in telomere elongation, allowing cells to preserve chromosomal integrity despite lacking telomerase.

The ALT mechanism is predominantly observed in tumors of mesenchymal origin, such as sarcomas, but is also frequently detected in glioblastomas. ALT functions through an HR-based process, facilitating the synthesis of new telomeric DNA by using an existing telomere as a template. This template may originate from a different chromosome’s telomere, another region of the same telomere via t-loop formation, or through recombination between sister telomeres. During this process, the 5′ overhang of a telomere can invade the T-loop of a homologous chromatid, forming a structure analogous to a replication fork. This structure is subsequently recognized by DNA polymerase, which extends the telomere, thereby maintaining telomere length in the absence of telomerase [2,6,10,11]. Several genetic alterations have been strongly associated with ALT activation. The most well-characterized mutations involve loss-of-function alterations in the Alpha Thalassemia/Mental Retardation Syndrome X-Linked (ATRX)/Death Domain-Associated Protein (DAXX) complex. ATRX deposits the histone variant H3.3 at pericentromeric and telomeric regions, helping to maintain heterochromatic integrity and suppress aberrant recombination; hence, it plays a crucial role in chromatin remodeling and telomere stability. The disruption of ATRX/DAXX results in the formation of G-quadruplexes and R-loops–non-B DNA structures that contribute to telomere instability and HR-driven telomere elongation [10,11,36]. Moreover, the absence of ATRX/DAXX triggers recombination-based repair at telomeres, thereby contributing to therapeutic resistance in ALT-positive cancers-particularly noted in gliomas and sarcomas’ therapy where conventional methods are ineffective [37,38,39]. Furthermore, ATRX/DAXX mutation was observed to increase aggressive behavior and risk of recurrence, as reported in studies regarding pancreatic neuroendocrine tumours. What is more, SMARCAL1 deficiency has been correlated with ALT activation, as cells lacking SMARCAL1 often exhibit ALT-associated promyelocytic leukemia bodies, which are hallmark features of the ALT pathway [10,40,41]. Additionally, mutations in TP53 (p53) have also been implicated in the induction of ALT, suggesting a broader role for tumor suppressor dysfunction in promoting telomerase-independent telomere maintenance [10,42,43]. Mutations in chromatin remodelling and genome stability genes-ATRX/DAXX, SMARCAL1, and TP53-promote ALT activation by enhancing telomeric recombination and disrupting heterochromatin maintenance. ALT mechanism is shown in Figure 4.

## 3. Telomere Maintenance and DNA Repair: A Bidirectional Relationship in Cancer Biology and Therapy

**Figure 5:**
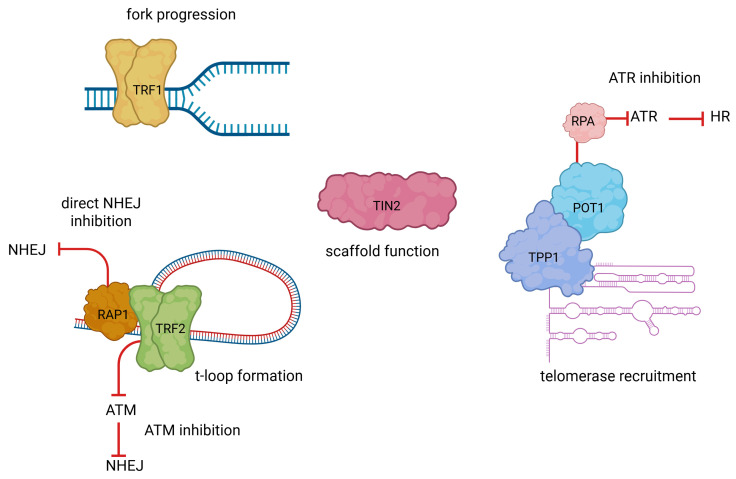
Roles of shelterin components in telomere replication and DNA damage response suppression. TRF1 promotes replication fork progression by facilitating the resolution of secondary DNA structures. TRF2 facilitates T-loop formation and suppresses NHEJ through inhibition of ATM signaling and recruitment of RAP1, which directly blocks NHEJ at telomeres. POT1, in complex with TPP1, inhibits HR by preventing ATR activation through sequestration of RPA-coated single-stranded DNA. TPP1 also enhances telomerase recruitment during S-phase. TIN2 serves as a central scaffold that stabilizes interactions among TRF1, TRF2, and the POT1–TPP1 complex to coordinate telomere protection and replication.

Telomere maintenance is critical for genomic stability, relying on a delicate interplay with DNA repair mechanisms. Importantly, this relationship is bidirectional: while telomere-bound proteins regulate DNA repair pathway activity, DNA repair enzymes also directly contribute to the preservation of telomere structure and function. The distinct roles of individual shelterin proteins in telomere protection and regulation of DNA damage response are illustrated in Figure 5. Shelterin’s pivotal function, primarily executed through TRF2-mediated formation of the T-loop structure, is to prevent DDR activation. This inhibition encompasses key signalling pathways such as ataxia–telangiectasia mutated (ATM) and the ataxia–telangiectasia and Rad3-related (ATR) kinases, as well as DNA repair processes such as non-homologous end joining (NHEJ) and HR. RAP1, recruited by TRF2, further safeguards telomeres by preventing fusions, particularly under conditions of t-loop instability. Beyond its protective role, shelterin is essential for telomere replication. TRF1 facilitates replication fork progression by recruiting helicases, such as Bloom syndrome protein (BLM), to resolve secondary DNA structures, mitigating replication stress in G-rich regions. Additionally, TIN2 acts as a structural scaffold, linking TRF1, TRF2, and the POT1–TPP1 complex to ensure coordinated telomere maintenance. During S-phase, shelterin regulates telomerase activity to counteract the end-replication problem. TPP1, via its TEL-patch, enhances telomerase recruitment, particularly at short telomeres, while POT1 suppresses excessive elongation. Following telomere extension, the CST complex (CTC1-STN1-TEN1) promotes C-strand fill-in synthesis by DNA polymerase α, thereby terminating telomerase activity. Beyond telomere-specific functions, certain shelterin components contribute to broader cellular processes. TRF1 plays a role in mitotic spindle regulation by modulating microtubule polymerization, whereas RAP1 influences gene expression, particularly in metabolic and inflammatory pathways. Emerging evidence also suggests a role for TIN2 in mitochondrial function and cellular aging. Collectively, shelterin ensures telomere stability and genomic integrity, preventing inappropriate DDR activation and maintaining chromosome end protection [44,45,46,47,48,49]. Recent studies have revealed non-canonical, stress-responsive functions of shelterin components in the DNA damage response. TRF2 transiently localizes to non-telomeric DSBs in a PARP1-dependent manner, colocalizes with DDR factors, and modulates HR and NHEJ pathways. It has also been implicated in mitochondrial regulation via SIRT3 interaction and in modulating immune responses relevant to cancer [50]. Under replication stress, POT1 mutations disrupt replication fork progression, activate ATR-mediated checkpoints, and promote telomere relocalization to the nuclear periphery through F-actin and nuclear pores, enhancing genome surveillance and repair [51].

**Figure 6:**
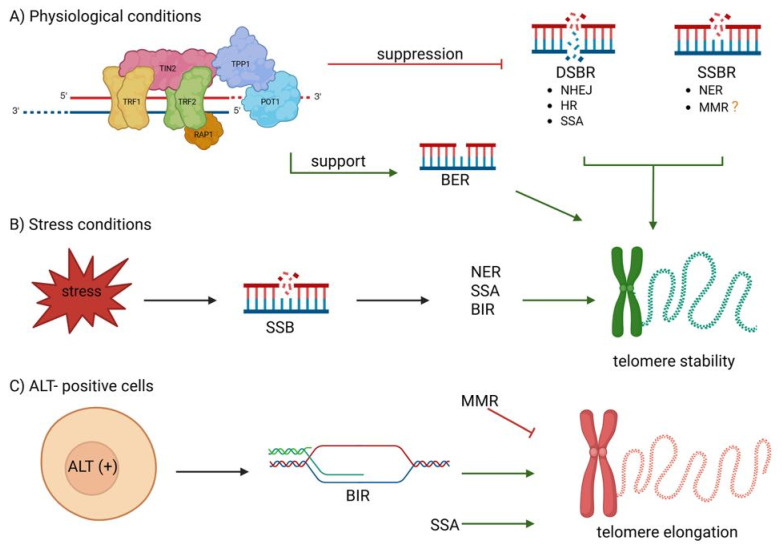
Overview of context-dependent interactions between DNA repair pathways and telomere regulation. (**A**) Under physiological conditions, shelterin promotes BER to repair oxidative base lesions while suppressing NHEJ, HR, SSA, and NER. The role of MMR at telomeres remains unclear. This selective suppression supports telomere stability. (**B**) Upon oxidative or replication stress, SSBs accumulate and trigger the recruitment of NER, BIR, or SSA to restore telomere stability. (**C**) In ALT-positive cells, telomeres are elongated through alternative homology-directed repair mechanisms, including BIR and SSA. Conversely, MMR acts as an anti-recombinase to limit excessive elongation.

Chromosomal ends structurally resemble DNA damage sites, necessitating the suppression of the DDR to prevent inappropriate repair that could lead to chromosomal fusions and consecutive genome destabilization. The DDR is orchestrated by three phosphatidylinositol 3-kinase-related protein kinases: ATR, ATM, and DNA-dependent protein kinase (DNA-PK). Among these, ATM and DNA-PK primarily respond to DSBs, while ATR is activated by replication stress and accumulation of single-stranded DNA. Multiple DNA repair pathways operate at chromosomal ends, with mechanisms tailored to different types of DNA damage. DSB repair pathways include NHEJ, HR, microhomology-mediated end-joining (MMEJ, also known as alternative NHEJ), break-induced replication (BIR), and single-strand annealing (SSA). Apart from DSB repair, other mechanisms address specific lesions, including nucleotide excision repair (NER), base excision repair (BER), mismatch repair (MMR), and direct repair (DR) [52,53,54]. DNA repair pathways are tightly regulated by shelterin and also contribute to telomere maintenance, forming a bidirectional relationship essential for chromosomal stability. Shelterin modulates multiple DNA repair pathways that, in turn, help preserve telomere integrity, establishing a bidirectional relationship crucial for chromosomal stability under physiological and stress conditions [52]. This bidirectional interplay is summarized in Figure 6.

NHEJ represents one of the main pathways that must be tightly suppressed at telomeres to prevent deleterious end-to-end fusions. Shelterin components achieve this by blocking key steps in the repair process. TRF2 promotes T-loop formation, which prevents the recruitment of KU70/80 and the MRN complex, thereby suppressing ATM activation. It also limits RNF168 and 53BP1 accumulation, further restricting NHEJ [55,56,57,58]. RAP1 reinforces this suppression by interacting with MRN, KU70/80, and PARP1, especially at critically short telomeres [53,59]. To inhibit A-NHEJ, the TPP1–POT1 complex blocks replication protein A (RPA) binding to single-stranded telomeric DNA, preventing activation of ATR-dependent repair [46,60,61]. Conversely, under conditions of telomere dysfunction, components of the NHEJ machinery, including DNA-PKs and KU70/80, can transiently associate with telomeres to stabilize chromosome ends and support end-capping, thereby limiting inappropriate DNA damage signalling [62,63].

HR is also actively restricted at telomeres. TRF2, in conjunction with RAP1, inhibits RAD51-mediated strand invasion, prevents homology search, and blocks cleavage of telomeric structures, such as T-loops, D-loops, and Holliday junctions. TRF2 also recruits BLM helicase to unwind recombination intermediates [57,60,61]. Additionally, POT1 suppresses HR by binding to telomeric ssDNA, preventing RPA and RAD51 from initiating strand invasion. Loss of POT1 results in excessive telomere resection, telomeric R-loops, and branched DNA structures [64]. Although HR must remain tightly restrained at telomeres, specific factors such as RAD51, XRCC3, and BRCA2 contribute to telomere maintenance by facilitating strand invasion, T-loop formation, and telomere replication. RTEL1, recruited by TRF2, ensures controlled disassembly of these structures [65,66].

Alternative homology-directed repair pathways such as BIR and SSA, alongside classical HR, actively shape telomere stability. In ALT-positive cells and in response to oxidative stress, TRF2 facilitates BIR by promoting R-loop formation through interaction with TERRA, thereby enabling RAD52-dependent telomere synthesis. This mechanism provides a telomerase-independent means of telomere maintenance but may also contribute to genomic instability [67,68]. BIR is both a cause and a consequence of replication stress, which must be tightly regulated to sustain ALT activity. Excessive stress can trigger hyperrecombination and cell death, while insufficient stress impairs ALT and leads to telomere shortening [69]. In contrast, SSA, which similarly relies on RAD52 but functions independently of RAD51, is generally suppressed at telomeres due to its propensity to induce large deletions and intrachromosomal rearrangements [54]. However, factors like ERCC1/XPF and SLX4 can be recruited to telomeres under stress. TERRA promotes XPF localization in FANCM-deficient cells, while phosphorylated SLX4 supports SSA and repair at ALT telomeres [54].

In addition, telomeres are also subject to single-stranded DNA lesions, which are typically repaired by pathways such as NER, BER, and MMR. While the efficiency of NER at telomeres remains debated, its deficiency leads to telomere fragility and loss, particularly under oxidative stress. NER removes helix-distorting lesions such as UV-induced photoproducts and bulky oxidative adducts that can block replication at telomeres. Shelterin components modulate NER activity, ensuring that repair is appropriately restricted or permitted depending on the extent of damage. Under basal conditions, TRF2 inhibits recruitment of the XPF-ERCC1 endonuclease complex, thereby preventing inappropriate cleavage at telomeric ends; however, this suppression can be relieved in response to severe DNA damage. Additionally, the transcription-coupled NER factor CSB associates with TRF2 to support telomere stability, while TFIIH has been implicated in TRF1-dependent telomere replication [56,57,70,71,72]. Loss or mutation of NER factors, such as XPF/ERCC1 or CSB, is linked to telomere dysfunction in progeroid syndromes, highlighting the pathway’s physiological importance [73].

Unlike other repair pathways that are suppressed at chromosome ends, BER supports telomere maintenance and is positively regulated by shelterin to counteract oxidative and alkylation-induced DNA damage. TRF2 enhances BER efficiency through direct interactions with Polβ and FEN1, facilitating strand displacement synthesis, while POT1 and TRF1 promote the activity of APE1 and DNA ligases. In addition, APE1 cooperates with TRF2 to mitigate oxidative stress, thereby preventing telomere shortening and cellular senescence [56,57,70,71,74]. Consistently, the loss of glycosylases such as OGG1 leads to increased telomere fragility under oxidative stress, underscoring BER’s critical role in preserving telomere integrity [75].

MMR contributes to telomere stability through distinct mechanisms involving its two major complexes. MutSβ has been implicated in preventing the formation of R-loops and the accumulation of G-quadruplex structures, both of which can compromise telomere integrity. In contrast, MutSα plays a role in repressing aberrant recombination events associated with ALT-mediated telomere maintenance. Still, the precise roles of MMR at telomeres remain only partially understood [54,56,71]. In ALT-positive cells, MSH2 and MSH6 are specifically recruited to telomeres, where MutSα functions as an anti-recombinase complex that limits uncontrolled telomere elongation. Loss of this activity leads to excessive telomere extension through ALT-associated homology-directed repair, underscoring the importance of MMR in preserving telomere homeostasis [76,77].

The interplay between shelterin and DNA repair pathways is not only essential for telomere homeostasis, but is also altered in cancer, enabling sustained proliferation and tolerance to genomic instability. Increasing evidence underscores the clinical relevance of telomere biology, with shelterin components emerging as promising therapeutic targets. Overexpression of TRF1 and TRF2 has been reported across multiple cancer types, where it correlates with increased cell proliferation (renal cell carcinoma) [78], advanced pathological features (prostate cancer) [79], and progressive upregulation during multistage carcinogenesis (gastric cancer) [80]. In contrast, POT1 mutations have been identified in angiosarcoma, implicating shelterin dysfunction in tumorigenesis, while in gliomas, such mutations may activate rare, non-canonical telomere maintenance mechanisms [81].

## 4. Therapeutic Targeting of Telomerase and ALT Pathways

Telomere maintenance is crucial for cellular immortality, the main advancement of cancer cells. Most malignancies achieve this by upregulation of telomerase. However, a subset of cancers employs the ALT pathway to maintain telomere integrity. Consequently, therapeutic methods of targeting these mechanisms have gained significant interest. Inhibition of telomerase activity in cancer cells leads to induction of telomere shortening and subsequent cellular senescence or apoptosis. Telomerase-related strategies can be categorized into oligonucleotide inhibitors, small-molecule inhibitors, immunotherapy, gene therapy, and naturally occurring substances [82,83]. Recent studies have highlighted the impact of these inhibitors on DDR pathways. Telomerase inhibition can trigger DNA damage response, activating mechanisms that may enhance therapeutic efficacy by sensitizing cancer cells to DNA damage-induced apoptosis [54].

### 4.1. Oligonucleotide-Based Inhibitors

To start with, oligonucleotide-based inhibitors, Imetelstat (GRN163L), are a 13-mer oligonucleotide conjugated to a palmitoyl group, which enhances cellular uptake. It binds with high affinity to the human telomerase (hTR) template region, acting as a direct competitive inhibitor of telomerase enzymatic activity. This prevents telomere elongation, leading to a reduction in telomere length and progenitor cell proliferation, and induction of apoptotic cell death. GRN163L has demonstrated potent inhibition of telomerase in multiple cancer cell lines, resulting in a reduction in cell proliferation and lifespan [84,85]. A non-lapidated version of Imetelstat, GRN163, also exhibits positive results in cancer cell therapy, but its effectiveness is lower due to decreased cellular uptake [86,87]. Imetelstat has been evaluated in multiple clinical trials for its safety and efficacy. In a recently conducted study, Kuykendall et al. and Mascarenhas et al. found that Imetelstat at 9.4 mg/kg administered every 3 weeks was safe and effective in relapsed/refractory myelofibrosis after JAK inhibitor failure. In the first study, Imetelstat use was associated with approximately a 65% reduction in mortality risk compared to standard treatment. In the latter, Imetelstat showed disease-modifying effects, including improvement in bone marrow fibrosis, reduced mutation burden, and diminished telomerase activity [88,89]. Conversely, Imetelstat failed as a maintenance therapy in a Phase II trial for advanced non-small-cell lung cancer, as no significant benefit was observed compared to the control group [90]. Overall, the current body of literature presents mixed findings regarding Imetelstat, and further investigation is warranted. Regarding GRN163, no clinical trials have been conducted to date.

### 4.2. Small Molecule Inhibitors

A group of small-molecule inhibitors also shows positive effects in cancer therapy. The non-nucleoside compound BIBR1532 represses telomerase activity by specifically binding to the conserved hydrophobic pocket (FVYL) motif in the active site of the human telomerase reverse transcriptase (hTERT), thereby inducing senescence in cancer cells [91,92]. It has exhibited broad anticancer effects in ovarian, chondrosarcoma, breast, lung, and germ cell tumors [93]. Another small molecule inhibitor, MST-312, a synthetic derivative of tea catechins, disrupts telomere length maintenance and induces G2/M cell cycle arrest, triggering DNA damage responses in various cancer cell lines, including lung, breast, and brain cancers. Additionally, MST-312 has been shown to inhibit angiogenesis and modulate immune responses, further contributing to its anticancer effects [94,95]. Although small-molecule inhibitors have proved their efficacy in preclinical studies, there have not been any clinical trials involving these compounds, highlighting the need for further research.

### 4.3. Immunotherapy

Immunotherapy that elicits immune responses against telomerase-expressing cells has emerged as a promising strategy. For instance, GV1001 is a 16-amino-acid peptide derived from the hTERT sequence, initially developed as a cancer vaccine. Beyond its immunotherapeutic role, GV1001 functions as a ligand for the gonadotropin-releasing hormone receptor (GnRHR), selectively stimulating the Gαs/cAMP signaling pathway while antagonizing Gαq-mediated calcium release. This mechanism inhibits prostate cancer cell proliferation and migration [96]. Moreover, GV1001 has been reported to induce apoptosis by reducing angiogenesis, further contributing to its anticancer potential [97,98]. A 2023 Phase 3 randomized trial demonstrated that GV1001 combined with gemcitabine/capecitabine in pancreatic cancer improved median overall survival (11.3 vs. 7.5 months) and time to progression (7.3 vs. 4.5 months) compared to chemotherapy alone [99]. Furthermore, ongoing studies suggest anti-inflammatory and anti-oxidative properties of GV1001 may enhance its utility across different solid tumors [100]. Another immunotherapeutic approach involves Vx-001, a peptide-based cancer vaccine designed to elicit a targeted immune response against tumor cells expressing hTERT. Clinical studies have shown that Vx-001 induces a robust hTERT-specific T-cell response, which is associated with prolonged survival in patients with advanced solid tumors. Notably, the efficacy of Vx-001 appears to be more pronounced in tumors with low or absent tumor-infiltrating lymphocytes (TILs), suggesting its potential as a therapeutic option for non-immunogenic tumors [101]. The study by Kotsiakis et al. demonstrated that Vx-001 was well tolerated and led to an increase in TERT-specific immunological response, with partial responses observed in 3 patients and disease stabilization in 23 others [102]. A Phase 2 trial by Gridelli et al. found that Vx-001 therapy was well tolerated, with no treatment-related toxicity greater than grade 2. While the trial did not meet its primary endpoint of improving overall survival in the entire study population, a subset of patients exhibited long-lasting TERT-specific immune response and experienced significantly longer median overall survival compared to non-responders (21.3 months versus 13.4 months, *p* = 0.004) [103].

### 4.4. Gene Therapy

Gene therapy represents a promising avenue of telomere-targeted cancer treatment. One notable strategy involves suicide gene therapy, in which therapeutic genes encoding cytotoxic proteins are selectively delivered to tumor cells. These genes are commonly regulated by the hTERT promoter, allowing for targeted expression specifically in telomerase-active cancer cells, while minimizing effects on healthy tissues. For example, a preclinical study on hepatocellular carcinoma utilized an adenoviral vector carrying a Tetrahymena group 1 trans-splicing ribozyme designed to target hTERT RNA. This approach effectively induced a selective cytotoxicity in telomerase-positive liver cancer cells at relatively low doses, with minimal hepatotoxicity observed in mouse xenografts [104]. These observations highlight the potential of telomere-targeted suicide gene therapy as an effective approach, warranting further investigation in future preclinical and clinical studies.

Another gene therapy approach involves oncolytic viruses, which are engineered to selectively replicate within and destroy cancer cells. Telomelysin (OBP-301), a telomerase-specific oncolytic adenovirus, utilizes the hTERT promoter to drive its replication, ensuring selective targeting of telomerase-positive tumor cells while sparing normal tissues. In a Phase I clinical trial, Telomelysin was evaluated in combination with radiotherapy for patients with esophageal cancer. The results were encouraging: 8 out of 13 patients achieved a complete response, 3 showed a partial response, and increased infiltration of CD8+ T cells was observed in the tumor area. The study reported an objective response rate of 91.7%, highlighting the potential of the combined therapeutic strategy [105]. Telomelysin demonstrated a favorable safety profile in advanced liver cancer with no dose-limiting toxicities up to 6 × 10^12^ viral particles. Disease stabilization was observed, as well as T cell infiltration and necrosis at the injection site, suggesting robust local immune activation [106]. These findings suggest that Telomelysin may offer significant local antitumor effects and hold promise for improved efficacy when used in combination with immunotherapeutic agents.

### 4.5. Naturally Occurring Compounds

Several naturally occurring compounds have been identified as telomerase inhibitors. Epigallocatechin gallate (EGCG), curcumin, camptothecin, berberine, and resveratrol exhibit inhibitory effects on human telomerase, leading to inhibition of cellular proliferation and induction of apoptosis. These substances have also been reported to modulate microRNA expression, thereby influencing oncogenic signaling pathways [107,108,109,110,111].

### 4.6. Targeting G4-Quadruplexes

Another way to influence the telomerase activity is by targeting specific nucleic acid structures called G4-quadruplexes. These are guanine-rich RNA or DNA non-canonical sequences being held by Hoogsteen hydrogen bonds [112]. They are not randomly distributed throughout the genome but are present within most human oncogenic promoters and telomeres. Genes that are most commonly associated with G4 are MYC protooncogene, the receptor tyrosine kinase, B cell lymphoma 2, VEGF, and KRAS. G4-quadruplexes are believed to have an impact on telomerase activity, protein expression, genome integrity, and cell killing [113,114].

Immunohistochemical investigations have demonstrated an elevated level of G4s in neoplastic tissues compared to normal tissues. Given that G4s influence genomic instability, an augmented intracellular concentration of these structures may serve as a potent catalyst for the evolution of the cancer genome, specifically the process of malignant transformation. These observations conclude that G4s play a pivotal role in carcinogenesis and the malignant phenotype of neoplastic cells. Based on the presented data, it is hypothesized that the pharmacological perturbation of G4 structural transitions offers a promising strategy for targeted oncological therapies [115].

The functional significance of G-quadruplex architecture is supported by identifying proteins capable of interacting with them and resolving these nucleic acid motifs. There are three groups of G4 ligands presented in the literature: attached to the surface of the G-quadruplex, inserted into the middle of the plane of the G-tetrad, and clung to the sides of the G-quadruplex [116]. To date, over 800 ligands have been found and described [115]. A wide range of chemical substances that interact with G4 structures has been documented. These include families of molecules based on acridine, pyridine, and porphyrin, as well as telomerase-blocking drugs and other varied compounds [116]. However, it is challenging to assess their binding preferences in living cells as they have been primarily tested in vitro, and some G4 ligands are also bound to other non-canonical forms. Nonetheless, G4-quadruplexes in telomeres were proven both in vivo and in vitro to block telomerase activity by modifying telomerase binding. That way was effective with Telometastatin, a natural product composed of seven oxazole rings and one sulfur-containing thiazoline [117], and with 2,6-diamidoanthraquinone derivatives. G4 ligand pentacyclic RHPS4 was also proven successful in inducing telomere instability via shelterin complex disruption [113,118]. Administration of G4 ligand RHPS suppresses a range of melanoma cells [119].

When creating new medications, the G-quadruplex presents a compelling target. Unlike the standard double-helix DNA, its unique, compact shape and distinct 3D arrangement open the door to designing exact drugs. This difference makes it possible to find molecules that can specifically interact with and reinforce or dismantle the G-quadruplex’s structure, all while leaving the far more prevalent helical DNA untouched. This selectivity is a key advantage in developing therapies that minimize unwanted interactions and side effects [118]. G4-targeting ligands are currently undergoing intensive preclinical evaluation, showing promising anticancer potential through disruption of telomerase function and oncogene expression, highlighting their future clinical application in targeted cancer therapies.

### 4.7. Inhibition of the ATR Kinase

Another telomere-associated strategy, particularly relevant to cancers utilizing the ALT pathway, involves the inhibition of the ATR kinase. ATR inhibitors such as VE-821 and Ceralasertib (AZD-6738) have been shown to selectively impair ALT-positive cells by inducing telomeric DNA damage and replication stress, leading to apoptosis [120,121]. However, emerging evidence suggests that ALT-positive tumor cells do not universally exhibit the hypersensitivity to ATR inhibition, indicating that the therapeutic efficacy of these agents may be influenced by additional cellular factors beyond telomere maintenance mechanisms [122]. Despite this variability, these findings support the potential of ATR inhibitors as a promising therapeutic option for targeting ALT-dependent malignancies. VE-821 was tested only in preclinical studies; nonetheless, Ceralasertib has shown promising effects in monotherapy or in combination with other therapeutic options. Dillon et al. reported that the RP2D for Ceralasertib is 160 mg once daily for 2 weeks in a 4-weekly cycle, whereas Kim et al. proposed the RP2D for Ceralasertib at a level of 240 mg twice daily on a 28-day schedule. Both study groups found Ceralasertib to be safe, and importantly, both study groups observed durable responses in responding patients [123,124]. Additionally, Kwon et al. found that Ceralasertib (dose 250 mg twice a day) alongside Durvalumab was safe, well tolerated, with manageable adverse effects, and achieved promising antitumor effects in patients with advanced gastric cancer. The study group observed an increased activation of intratumoral lymphocytes and expansion of circulating tumor-reactive CD8+ T cell clones in responding patients, along with improved overall response rate, disease control rate, and median progression-free survival [125]. Moreover, the combination of Ceralasertib with Durvalumab was found to be effective in advanced/metastatic melanoma. As reported by Kim et al., among 30 patients, almost ⅓ exhibited an overall response, and over 60% showed disease stabilization. Ceralasertib was well tolerated, with manageable toxicity (dose 240 mg twice a day) [126].

### 4.8. RAD51 Inhibitors

Complementing ATR inhibition, another emerging strategy for targeting the ALT mechanism in cancer relies on inhibition of RAD51, which facilitates DNA strand invasion and exchange during the process of HR. By blocking RAD51, the whole process of telomere maintenance in cancers might be disrupted. One study group observed three responses in patients with relapsed/refractory hematologic malignancies and advanced solid tumors with the CYT-0851-RAD51 inhibitor. Moreover, only mild side effects were reported, showing the potential of this compound [127]. Furthermore, ri-1 and BO2, other RAD51 inhibitors, were shown to improve outcome in a few preclinical studies. For example, Chen et al. reported suppressed cervical cancer cell proliferation with the administration of this ri-1, whereas King et al. observed a reduction in double-strand break repair in glioblastoma stem cells that led to significant radiosensitization [128,129]. However, further research is needed for a full assessment.

### 4.9. CHK1 Inhibitors

Expanding on the theme of DDR targeting, inhibition of CHK1, a key modulator of cell cycle checkpoints and HR, has also gained attention. CHK1 serves as a pivotal node connecting DNA damage sensing with repair pathways, making it an attractive target for cancer therapy [130,131]. Among notable agents, Prexasertib has shown clinical activity, particularly when combined with PARP inhibitors. Khanh T Do et al. reported in phase 1 preliminary clinical data that combining CHK1 inhibitor Prexasertib with the PARP inhibitor Olaparib shows therapeutic promise in patients with high-grade serous ovarian cancer (HGSOC) who carry Breast Cancer Gene (BRCA) mutations and have experienced disease progression despite prior PARP inhibitor treatment. Pharmacodynamic analyses indicate that Prexasertib’s mechanism of action involves impairing HR, thereby inducing DNA damage and replication stress within the cancer cells. However, some adverse effects like leukopenia, neutropenia, thrombocytopenia, and anemia have been noted among the patients [131]. BBI-355 is a newer, oral, selective CHK1 inhibitor with a specific focus on cancers with oncogene amplifications, particularly those on extrachromosomal DNA (ecDNA). Preclinical data presented by Brad et al. show significant synergistic anti-tumor activity when BBI-355 is combined with targeted therapies (like EGFR or FGFR inhibitors) in ecDNA-positive, oncogene-amplified gastric and esophageal cancers. This targets a specific subset of difficult-to-treat cancers. Initial clinical trials of BBI-355, both as a monotherapy and in combination with Erlotinib or Futibatinib, revealed generally manageable dose-limiting hematological toxicities. Clinical testing in patients with EGFR and FGFR2 oncogene amplifications is still ongoing [132].

A comprehensive overview of the mentioned compounds, additionally including their clinical trial key findings regarding their efficacy in studies, is provided in Table 1, whereas their telomere-related applications in oncology are presented in Table 2.

## 5. Challenges in Targeting Telomere Dynamics for Cancer Therapy

### 5.1. Diagnostic and Prognostic Applications

Previous work has already established that analyzing tumor cell telomeres is helpful in the scope of biomarker research to determine tumor malignancy and therapeutic potential. Using a prognostic model based on 18 telomere-related genes, it is feasible to distinguish between low- and high-risk non-small cell lung cancer (NSCLC) patients, and telomere-related gene expression is closely related to patients’ overall survival. Interestingly, this model was linked to immune-related pathways and was able to predict the response to PD-L1 blockade therapy while highlighting the role of telomere maintenance in the tumor microenvironment [137].

A similar study on telomere-related genes and relative telomere length (TL) of cfDNA suggested its potential as a biomarker for endometrioid endometrial cancer (EEC) with high sensitivity and specificity [138]. A similar approach can be used to follow up the treatment outcomes in patients with breast cancer [145]. cfDNA telomere length was found to decrease after neoadjuvant chemotherapy, especially in patients with a good response to treatment, while telomere lengths remained stable in non-responders or metastatic patients [139]. This method is attractive as it entails the easy collection of cell-free DNA from the blood.

Telomere-based monitoring is also applied in epithelial ovarian cancer (OvC). TL was quantified in peripheral blood leukocytes (PBL), tumor samples from 209 OvC patients using multiplex qPCR, and methylation and gene expression of the shelterin complex and hTERT were examined. The results indicated that PBL TL was shorter in chemotherapy-sensitive patients than in treatment-refractory patients. PBL TL may be a promising predictive factor for the response to platinum-based therapy, while the TL of the tumor may contribute to the overall survival of patients with OvC [140].

### 5.2. Prognosis, Drug Response, and Therapeutic Targets

A positive correlation between telomere length in cancer cells and cancer-associated fibroblasts (CAFs) has also been observed in adenocarcinoma. Cancers with desmoplastic stroma, including scirrhous adenocarcinoma, had longer telomeres in cancer cells and CAFs. The results of this study indicated that overall survival was worse with longer telomeres in these cells, and CAF TL was linked to worse DFS. Moreover, TL variations depended on age, sex, and histological grade, with advanced-stage and highly malignant tumors exhibiting longer telomeres [141,146].

Furthermore, telomere biology may serve as a predictive marker for adverse drug reactions (ADR). For instance, in NSCLC, shorter relative telomere length (RTL) in blood leukocytes has been associated with a higher risk of Osimertinib-related adverse drug reactions. This suggests that relative TL could be a promising biomarker for determining the severity of ADRs to Osimertinib in patients with advanced-stage NSCLC [142].

The prediction of treatment outcomes for ALT tumors remains a challenge. The presence of extrachromosomal telomeric DNA—C-circles—appears to be a superior marker of ALT activity. These C-circles are 1000-fold more abundant in ALT cancer cells than in telomerase-positive tumor cells or normal cells, making them an essential diagnostic tool. Despite their reliance on ALT for telomere maintenance, these tumors are often more resistant to telomerase-targeted therapies. For example, in osteosarcoma, the absence of a detectable telomere maintenance mechanism is associated with better overall survival. In some cancers, such as diffuse malignant peritoneal mesothelioma, telomerase activity correlates with poor prognosis, whereas ALT status does not significantly impact patient survival [147]. Recently, ALT tumors have been shown to protect C-circles from nuclease degradation in the blood by releasing them within exosomes, which may serve as a novel blood-based biomarker for the detection and monitoring of ALT tumors in vivo [144,148]. In conclusion, non-invasive methods, such as liquid biopsy detection of C-circles or telomeric DNA in exosomes, may further expand the utility of telomere biomarkers for early diagnosis and monitoring of treatment response [149].

### 5.3. Challenges in Telomere-Based Therapeutics

Telomere therapy can be beneficial in many cases, but the most significant challenge is to avoid off-target effects. Normal human cells also have telomeres that prevent premature aging. However, cancer cells are characterized by much longer telomeres, making them a good treatment target. One of the most widely used strategies to inhibit cancer growth is the downregulation of hTERT [150]. It was established that suppressing ULK1, a key autophagy regulator, decreases hTERT activity and shortens telomeres in cancer cells, inducing cellular senescence. However, the same process takes place in non-cancerous liver cells, which may lead to senescence and increase the risk of age-related diseases such as degenerative disorders [151]. Furthermore, the inhibition of autophagy affects hTERT expression and cellular lifespan through the IL-6/STAT3 signalling pathway.

Another issue in drug development is that some types of cancer have developed certain compensatory mechanisms that allow cancer cells to activate alternative pathways, thereby escaping senescence and apoptosis. The ALT has been observed in high-risk neuroblastoma and certain sarcomas, and ALT-positive cancers present a serious clinical challenge with no known targeted therapies. Recent studies show that APR-246, a p53 reactivator, is effective against ALT tumors in mice and that, when combined with irinotecan, it shows efficacy in ALT-positive models [143].

## 6. Summary and Future Perspectives

Telomere maintenance is essential for genomic stability, primarily through the shelterin complex (TRF1, TRF2, POT1, TIN2, TPP1, RAP1), which protects telomeres from DNA damage recognition and regulates telomerase activity. TRF2 promotes t-loop formation to suppress ATM, ATR, and NHEJ pathways, while RAP1 prevents telomere fusions. Shelterin proteins also modulate DNA repair pathways—TRF2 inhibits HR and NHEJ but promotes BIR in ALT-positive cells, and BER counters oxidative damage at telomeres.

Telomere biology is emerging as a cornerstone in the next generation of cancer therapeutics. A growing number of compounds are currently under investigation, highlighting the potential of telomere-based therapies to revolutionize cancer treatment. Many of these agents have already shown promising results in preclinical studies and are demonstrating clinical potential, for example, Imetelstat, immunotherapies such as GV1001 and Vx-001, Ceralasertib, CHK1 inhibitors, and natural compounds including EGCG, Camptothecin, curcumin, resveratrol, and berberine. However, several candidates, such as small molecule inhibitors, G-quadruplex stabilizing agents, ATR inhibitors like VE-821, or RAD51 inhibitors, have yet to be evaluated in clinical trials, underscoring the critical need for further research to fully assess their safety and efficacy. Although telomere-based therapies hold great potential, minimizing off-target effects to prevent premature aging in normal cells remains challenging.

Telomere-based biomarkers are under investigation for cancer prognosis and treatment response. TL and telomere-related gene expression correlate with progression and therapy outcomes in cancers such as NSCLC, breast, ovarian, and endometrial cancers. Shorter telomeres may predict better chemotherapy response, while longer telomeres in CAFs indicate poor prognosis.

These data show that telomere-related biomarkers could play a central role in the personalization of cancer therapy, leading to improved patient outcomes. Telomere-related genes, telomere length, and ALT activity are evaluated to serve as diagnostic, prognostic, and predictive tools that could enable dynamic, real-time treatment stratification to tailor therapeutic approaches for individual telomere biology with accuracy. Future studies should aim to refine telomere-targeting strategies and to develop telomere-related biomarkers to facilitate earlier and more precise personalization of treatment.

## 7. Conclusions

Telomere maintenance mechanisms play a pivotal role in cancer progression, with telomerase upregulation and ALT pathway activation enabling uncontrolled cell proliferation. This review highlights the bidirectional relationship between telomeres and DNA repair pathways, both essential for genomic stability. It also presents the current landscape of telomere-targeted therapies—including oligonucleotides, small molecules, immunotherapy, gene therapy, and natural compounds. Although these approaches show promise, challenges such as biological complexity, adverse effects, and therapeutic resistance remain. Ongoing research in this field is vital and holds significant potential to reshape future cancer therapies.

## Figures and Tables

**Table 1:** Summary of Clinical and Preclinical Trials Targeting Telomerase and ALT Pathways in Cancer Treatment.

Therapeutic Strategy	Compound	Therapeutic Action	Key Clinical Outcomes	Reference
**Telomerase-related strategies**				
Oligonucleotide-based inhibitors	Imetelstat (GRN163L)	Inhibits hTR, induces telomere shortening and apoptosis	Imetelstat might be beneficial in the therapy of relapsed/refractory myelofibrosis	[84,85,88]
	GRN163	Similar to GRN163L, less effective due to reduced uptake		[86,87]
Small molecule inhibitors	BIBR1532	Binds FVYL motif in hTERT; blocks telomerase, induces senescence	There are no current clinical trials	[91,92]
	MST-312	Telomere shortening; G2/M arrest and DDR activation	There are no current clinical trials	[94,95]
Natural telomerase inhibitors	EGCG	Telomerase inhibition; apoptosis induction	Phase 2 trial; EGCG was well tolerated and slightly reduced the recurrence of colonic neoplasia (29% compared to 35% in placebo)	[111]
	Camptothecin	Telomerase inhibition; DNA damage response	Although camptothecin itself was limited due to toxicity, a Phase 2 trial evaluating its derivative, irinotecan, in combination with bevacizumab demonstrated promising activity in patients with recurrent glioblastoma	[108,133]
	Curcumin	Telomerase inhibition; anti-oncogenic signalling	Phase 2 combining curcumin with paclitaxel in metastatic breast cancer patients showed a higher objective response rate compared to paclitaxel with placebo	[107,134]
	Resveratrol	Induces apoptosis and telomerase inhibition	Promising preclinical trials; definitive clinical efficacy results are pending	[110]
	Berberine	Telomerase inhibition; miRNA modulation	Phase 3 trial; berberine was well tolerated and reduced the recurrence rate of colorectal adenomas compared to placebo (36% vs. 47%)	[109,135]
Immunotherapeutic telomerase vaccines	GV1001	Anti-proliferative via GnRHR signalling; reduces angiogenesis	Phase 3 trial; combination therapy of GV1001 with chemotherapy in pancreatic cancer improved median overall survival and prolonged time to disease progression compared to chemotherapy alone	[97,98,99]
	Vx-001	Triggers hTERT-specific T-cell response	Phase 2 trial; median overall survival in patients with non-small cell lung cancer was increased	[101,103]
Telomerase-targeted gene therapies	Suicide gene therapy	Cytotoxic gene proteins are selectively delivered to tumor cells. These genes are commonly regulated by hTERT promoter, allowing for targeted expression specifically in telomerase-active cancer cells.	There are no current clinical trials	[104]
	Telomelysin	Oncolytic viruses are engineered to selectively replicate within telomerase-positive tumor cells and destroy cancer cells	Phase 1 clinical trials showed that Telomelysin was well-tolerated and significantly improved the results	[105,106,136]
**Inhibition of ALT**				
G4 quadruplex stabilizing agents	Telomestatin	Stabilizes telomeric G4 structures; inhibits telomerase	Preclinical trials; promising results in vitro but not advanced to clinical trials due to challenges in synthesis and stability	[117]
	RHPS4	Induces telomere stability via shelterin disruption	There are no current clinical trials	[113,118]
ATR inhibitors	VE-821	Induce replication stress and telomeric DNA damage in ALT cells	There are no current clinical trials	[120,121]
	Ceralasertib (AZD-6738)		Ceralasertib has demonstrated safety and well-tolerance along with anti-tumor response in a few clinical studies	
RAD51 inhibitors	CYT-0851	Blockage of HR process that ALT cells rely on to elongate telomeres	Phase 1 study showed responses and well-tolerance with manageable side effects, with improved	[127]
	ri-1		Showed promising results in preclinical studies but there are no current clinical trials	[128,129]
	BO2			
CHK1 inhibitors	Prexasertib	Impairing HR, thereby inducing DNA damage and replication stress within the cancer cells	Phase 1 clinical trials showed Prexasertib, in combination with Olaparib, shows therapeutic promise in patients with HGSOC who carry BRCA mutations and are PARP inhibitor-resistant	[131]
	BBI-355	Prevents cancer cells from properly managing and repairing DNA damage and replication stress	Preliminary clinical trial data suggest that the treatment is well tolerated	[132]

**Table 2:** Summary of telomere-based applications in oncology.

Application Area	Cancer Type	Biomarker/Target	Key Findings	Method	Clinical Relevance	Telomere Connection (Targeted Molecule/Pathway)	Reference
Prognostic model	NSCLC	18 telomere-related genes	Distinguishes high/low risk; predicts survival and PD-L1 therapy response	Gene expression profiling	Helps personalize immunotherapy	Telomere-related gene expression	[137]
Diagnostic biomarker	EEC	cfDNA relative TL	High sensitivity/specificity for diagnosis	Blood-based cfDNA measurement	Non-invasive early detection	Relative TL in cfDNA	[138]
Treatment monitoring	Breast cancer	cfDNA TL	Decreases post-chemotherapy in responders	Liquid biopsy	Tracks treatment response	cfDNA TL fluctuation	[139]
Predictive biomarker	Ovarian cancer	PBL TL, tumor TL	Shorter PBL TL = better chemosensitivity	qPCR + gene/methylation analysis	Predicts therapy response	Peripheral blood and tumor TL	[140]
Prognostic marker	Adenocarcinoma	TL in cancer cells and CAFs	Longer TL = worse prognosis	Tissue-based TL analysis	Correlates with survival	TL in cancer cells and fibroblasts	[141]
Adverse drug reaction predictor	NSCLC	Blood leukocyte TL	Shorter TL = higher risk of ADRs to Osimertinib	Leukocyte TL measurement	Patient safety stratification	TL as an indicator of drug tolerance	[142]
Therapeutic strategy	Various	hTERT	Imetelstat effective; manageable toxicity	Oligonucleotide therapy	Treats hematologic and solid tumors	Direct hTERT inhibition	[84]
Therapeutic target	ALT-positive tumors	p53 via APR-246	Effective in combination with irinotecan	Drug combination therapy	Promising for ALT+ cancers	ALT pathway and p53 restoration	[143]
Diagnostic tool	ALT tumors	C-circles in exosomes	ALT-specific and stable in blood	Liquid biopsy	Non-invasive monitoring of ALT activity	ALT-specific telomeric DNA circles (C-circles)	[144]

## Abbreviations

ADR	Adverse Drug Reactions
ALT	Alternative Lengthening of Telomeres
ATM	Ataxia–Telangiectasia Mutated
ATR	Ataxia–Telangiectasia And Rad3-Related Kinases:
ATRX	Alpha Thalassemia/Mental Retardation Syndrome X-Linked
BER	Base Excision Repair
BIR	Break-Induced Replication
BLM	Bloom Syndrome Protein
BRCA	Adverse Drug Reactions
CAF	Cancer-Associated Fibroblasts
CST complex	Ctc1–Stn1–Ten1 Complex
DAXX	Death Domain-Associated Protein
DDR	Dna Damage Response
DNA-PK	Dna-Dependent Protein Kinase
DR	Direct Repair
DSB	Double-Strand Break
EBV	Epstein-Barr Virus
ecDNA	Extrachromosomal Dna
EEC	Endometrioid Endometrial Cancer
EGCG	Epigallocatechin Gallate
FVYL	Conserved Hydrophobic Pocket (Phenylalanine/Valine/Tyrosine/Leucine)
GnRHR	Gonadotropin-Releasing Hormone Receptor
HGSOC	High-Grade Serous Ovarian Cancer
HL	Hodgkin Lymphoma
HR	Homologous Recombination
hTERT	Human Telomerase Reverse Transcriptase
hTR	Human Telomerase
MMEJ	Microhomology-Mediated End-Joining
MMR	Mismatch Repair
MRN	Mre11–Rad50–Nbs1 Protein Complex
NER	Nucleotide Excision Repair
NHEJ	Non-Homologous End Joining
NSCLC	Non-Small Cell Lung Cancer
OvC	Ovarian Cancer
POT1	Protection of Telomeres 1
RAP1	Repressor/Activator Protein 1
RP2D	Recommended Phase 2 Dose
RPA	Replication Protein A
SSA	Single-Strand Annealing
TERC	Human Telomerase Rna
TERT	Telomerase Reverse Transcriptase
TIL	Tumor-Infiltrating Lymphocytes
TIN2	Trf-Interacting Nuclear Protein 2
TL	Telomere Length
TPP1	Tripeptidyl-Peptidase 1
TRF1	Telomeric Repeat-Binding Factor 1
TRF2	Telomeric Repeat-Binding Factor 2
